# Cocktail-style multifunctional resuscitation fluids: Where are we now in 2026?

**DOI:** 10.1016/j.jatmed.2026.02.001

**Published:** 2026-03-20

**Authors:** Geoffrey L. Liu, Lauren C. Hollifield, Frank Chen, Henry Liu

**Affiliations:** a Perelman School of Medicine, University of Pennsylvania, 3401 Spruce Street, Philadelphia, PA 19104, USA; b Department of Anesthesiology, University of Texas Southwestern Medical Center, 5323 Harry Hines Boulevard, Dallas, TX 75390, USA

**Keywords:** Resuscitation, Multifunctional fluids, Shock, Hemorrhage, Sepsis, Oxygen carrier

## Abstract

For over half a century, fluid resuscitation has traditionally included crystalloids for volume expansion and colloids for potential oncotic advantage. The paradigm, however, could be shifting now. The development of multifunctional resuscitation fluids is fast evolving. These fluids integrate multiple therapeutic components. Rather than serving as simple volume expanders, they function as advanced therapeutic platforms engineered to simultaneously target multiple pathological conditions. They correct hypovolemia, mitigate systemic inflammation and coagulopathy, improve organ perfusion and oxygen delivery, and support tissue recovery in conditions such as shock, endothelial dysfunction, and oxidative stress. This article reviews the basic concepts underpinning multifunctional resuscitation fluids and explores their potential clinical applications. It also assesses their advantages and limitations based on recent preclinical and early clinical trials, and outlines future directions necessary to translate these promising solutions from the bench to the bedside.

## Introduction

Resuscitation fluids are an essential part of managing patients with acute hypovolemia, sepsis, trauma, burns, or shock, as well as in major surgical procedures with potential massive bleeding, as in liver transplantation and major cardiovascular surgery. The foundational goals of resuscitation are restoring adequate tissue perfusion and oxygen delivery, and mitigating the endothelial dysfunction and coagulopathy as well as maintaining hemodynamic stability.^1^, ^2^ Current fluid management mostly using 0.9% saline, Lactated Ringer’s, and Plasmolyte to primarily achieve volume expansion, albumin and other colloids to maintain oncotic pressure. However, these resuscitation fluids and allogeneic blood transfusion may worsen outcomes because they have been reported to cause various side effects, such as hemodilutional anemia, thrombocytopenia, coagulopathy, volume overload, and negative immunomodulation.^3^ These fluids are pharmacologically “inert” and do not mitigate the cellular and molecular injuries that accompany circulatory shock. The concept of multifunctional resuscitation fluids (MRF) emerged from the need to move beyond simple volume replacement to active resuscitation, where the fluid itself acts as a drug delivery system and/or a therapeutic agent.^1^, ^2^, ^3^, ^4^, ^5^, ^6^ Reynolds et al. reported adding a large molecular weight polymer of cross-linked bovine hemoglobin (Hb), OxyVita by OXYVITA Inc, New Windsor, NY, to a hypertonic saline and Hetastarch resuscitation protocol as an alternative to standard small-volume resuscitation using Hetastarch only. They found that small-volume resuscitation with OxyVita improved animal survival significantly and also improved mean arterial blood pressure. These results suggested that OxyVita-augmented hypertonic cocktails are promising in improving survival and maintaining targeted mean arterial blood pressure during small-volume resuscitation.^4^ Dickson et al. attempted combining Hetastarch with vasopressin to treat severe polytrauma in swine model with traumatic brain injury, hemorrhagic shock and simultaneous femur fracture by applying damage control resuscitation.^5^ However, the physiological status and their survival of experimental animals in both studies were significantly affected by the severity of hemorrhage regardless of resuscitation treatment. MRFs emerged as a promising strategy to target multiple physiological alterations simultaneously, driven by advancements in nanotechnology, synthetic biology, and a deeper understanding of hypovolemic and shock pathophysiology.^6^ However, translating these experimental solutions into routine clinical practice has faced several challenges, from formulation optimization to patient-specific response variability. This article aims to provide a comprehensive review of the current status of the MRF development.

## Key components and functions

MRFs aim to go beyond simple crystalloid and colloid solutions, which may have limitations in patients with severe hemorrhage, metabolic disturbances, and coagulopathy. The cocktail-style MRFs are designed to address these multiple physiological disturbances/failures that occur during severe bleeding and other critical clinical conditions simultaneously.^1^, ^2^, ^3^, ^4^, ^5^, ^6^ MRFs should include the following key components offering oxygen-carrying capability, hemostasis, hemodynamic support, volume expansion, anti-inflammatory and metabolic improvement.

### Oxygen transport enhancement

Oxygen-carrying components of MRFs may include one or more of the following elements.

#### Hemoglobin-based oxygen carriers (HBOCs)

Human, bovine, pig, engineered human, or recombinant hemoglobin is encapsulated within phospholipid bilayers (liposomes, mimicking red blood cells), or conjugated to polymers like polyethylene glycol (PEG).^7^, ^8^ This design of HBOCs minimizes nitric oxide scavenging and hemoglobin toxicity, while effectively carrying oxygen.^8^

#### Perfluorocarbon-based oxygen carriers (PBOC)

The addition of PBOCs into MRFs is under investigation for treating severe blood loss, especially in trauma and combat scenarios. PBOCs act as a synthetic alternative to transfusion of red blood cells, offering high oxygen solubility and anti-inflammatory properties, thus enhancing oxygen delivery.^9^

#### Nanoparticles

Nanotechnology is used to design HBOCs that can safely and effectively deliver oxygen to organs and tissues in human body, potentially including those tissues that natural red blood cells cannot reach.^10^

### Hemostasis/correction of coagulopathy

MRFs can have hemostatic effects by adding pharmacological or biological components, which will potentially correct coagulopathy by replacing lacking clotting factors or elements. Fibrinogen, Vitamin K, tranexamic acid and/or specific reversal agents can be added to MRFs.^11^ The more specific treatment will depend on the underlying cause of the coagulopathy, such as liver disease or anticoagulant use. However, fresh frozen plasma, cryoprecipitate, prothrombin complex concentrate and recombinant factor (rF)VIIa cannot be added to MRFs in the same fluid bag or intravenous line.^11^

#### Platelet mimics

Synthetic nanoparticles are being developed to mimic the function of natural platelets and help forming blood clots. These are designed to be freeze-dried and can be reconstituted on demand.^12^

#### Fibrinogen and Tranexamic acid

Both are studied as components of experimental low-volume resuscitation cocktails. Fibrinogen and tranexamic acid have been incorporated into to MRFs to improve hemostasis and reduce blood loss.^13^

#### Vitamin K

It is used for patients with a risk of vitamin K deficiency, such as those with malnutrition or cholestasis, to aid in the production of clotting factors.^14^

However, specific anticoagulant reversal agents like Andexanet alfa, the specific reversal agent for factor Xa inhibitors (rivaroxaban, apixaban, edoxaban), and Idarucizumab, the specific reversal agent for direct thrombin inhibitor (dabigatran), are not recommended to add to MRFs in the same bag.^11^

### Volume expansion and hemodynamic support

#### Colloid plasma expanders

Colloid plasma expanders will be used in MRFs to restore blood volume more effectively and for a longer duration than crystalloid fluids, which usually quickly leak out of the intravascular space. However, hypertonic saline can potentially create an osmotic gradient that draws fluid from the interstitial space into the circulation, allowing for rapid volume expansion with a smaller infused volume. In patients with traumatic brain injury (TBI), it can also help reduce intracranial pressure.^15^

#### Vasopressors

Some cocktails may contain agents like vasopressin to help maintain vital organ perfusion by keeping appropriate blood pressure levels during prolonged resuscitation.^16^

### Anti-inflammatory effects

MRFs may include endothelium-stabilizing and anti-Inflammatory agents, thus MRFs can provide some protection to the vascular endothelium, the primary casualty in circulatory shock.

#### Hyaluronic acid-containing MRFs

A hyaluronic acid-containing MRF is a type of intravenous fluid designed to not only restore blood volume but also repair the degraded endothelial glycocalyx. The endothelial glycocalyx is a delicate, gel-like layer that lines the inside of blood vessels and is often damaged during critical illnesses like trauma, sepsis, and hemorrhagic shock. Restoring this protective layer is a key target for improving patient outcomes.^17^

#### Sphingosine-1-Phosphate (S1P) receptor

Sphingosine-1-Phosphate (S1P) receptor agonist-containing fluids are an emerging area of research for treating conditions that involve endothelial damage, such as hemorrhagic shock, sepsis, and ischemia-reperfusion injury. By promoting endothelial barrier integrity, these fluids aim to reduce dangerous leakage of fluids from blood vessels, which can lead to tissue swelling (edema) and organ damage. Endothelial cells, which form the inner lining of blood vessels, rely on strong cell-to-cell junctions to regulate the passage of fluids and solutes. In conditions like shock or severe injury, these junctions break down, thus increasing vascular permeability. Sphingosine-1-Phosphate is a bioactive lipid that helps maintain the stability of these junctions by signaling through its receptors on endothelial cells.^18^, ^19^

#### Anti-oxidant infusions

MRFs containing molecular scavengers like Tempol or agents that boost endogenous glutathione production are being explored to treat conditions involving severe oxidative stress. This novel approach combines traditional fluid resuscitation with targeted therapeutic agents to address the underlying cellular damage caused by ischemia-reperfusion injury, sepsis, and other trauma-related conditions.^20^

Pharmacologic “cargo-carrying” MRFs are advanced, nanoparticle-based platforms that serve as innovative fluids, acting as controlled-release systems for targeted drug delivery to address specific complications in critical conditions like severe hemorrhagic shock or cardiac arrest. Examples include: Liposomes or Polymersomes loaded with antibiotics (for septic shock), hemostatic agents (for traumatic hemorrhage), or adenosine receptor agonists (to precondition tissues against ischemia-reperfusion injury).^21^

Buffering and metabolic resuscitation fluids are modern intravenous solutions designed to not only replace lost fluid volume but also help correct systemic metabolic disturbances, especially acidosis. This provides a significant advantage over older, single-purpose fluids like normal saline (0.9% sodium chloride) or Lactated Ringer’s solution. Moving beyond lactate, these fluids contain alternative buffers (e.g., pyruvate, ketone bodies) that also serve as superior metabolic substrates for ischemic tissues, potentially improving cellular bioenergetics and reducing lactic acidosis.^22^, ^23^ The mechanistic rationales, research and development stages, and their pertinent validating literature are summarized in Table 1.

**Table 1:** Components of modern resuscitation fluids.

**MRF components**		**Mechanistic rationales**	**R & D stages**	**Pertinent studies**
O_2_ carrying agents	HBOCs	Restore oxygen delivery independent of erythrocytes; scavenge nitric oxide, modulate microvascular perfusion	Preclinical complete; Advanced clinical trials ongoing. Pending FDA approval	Increased oxygen delivery but side effects concerning. Potential ↑risk of MI and mortality in meta-analyses^8^
	PBOCs	Dissolve and transport oxygen physically; enhance O_2_ diffusion in hypoxic microcirculation	Perftoran approved in Russia and South Africa. Clinical trials ongoing	Improved tissue oxygenation in surgical and experimental shock models^9^
Hemostatic agents	Platelet mimics	Bind exposed collagen and fibrin; promote platelet-like aggregation and clot stabilization	Clinical trials ongoing	Reduced bleeding and improved survival in hemorrhagic shock models^12^
	Fibrinogen	Restores clot firmness; enhances fibrin polymerization in trauma-induced coagulopathy	Phase II–III clinical; approved in many regions	Early fibrinogen replacement improves clot strength and reduces transfusion^13^
	Tranexamic acid	Inhibits plasmin-mediated fibrinolysis; stabilizes formed clots	Approved; phase IV outcomes research	Reduced mortality when administered early after trauma or postpartum hemorrhage^13^
	Vit K	Restores γ-carboxylation of clotting factors II, VII, IX, X	Approved; standard therapy	Effective reversal of vitamin K antagonist–associated coagulopathy^14^
Volume expanders	Colloids (albumin, hydroxyethyl starch)	Expand plasma volume via oncotic pressure; modulate endothelial permeability	Approved; ongoing safety evaluation	Albumin comparable to saline; synthetic colloids associated with renal injury^15^
Hemodynamic support	Vasopressin	V1 receptor–mediated vasoconstriction; restores vascular tone in catecholamine-resistant shock	Approved; Clinical research ongoing	Reduced norepinephrine requirements in septic shock; lower mortality benefit overall^16^
Antiinflammatory agents	Hyaluronic acid	Preserves endothelial glycocalyx; reduces vascular permeability and leukocyte adhesion	Clinical trials ongoing	Attenuates endothelial injury in hemorrhagic and septic shock models^17^
	Sphingosine-1Phosphate	Enhances endothelial barrier integrity; limits capillary leak	Clinical trials ongoing	Reduced vascular leak and organ injury in experimental shock^18^, ^19^
	Anti-oxidant	Scavenge reactive oxygen species; restore endothelial nitric oxide signaling	Clinical trials ongoing	Improving short-term mortality in patients with sepsis, but the evidence^20^
Metabolic correction	LR solution; Plasmolyte	Correct metabolic disturbances; reduce chloride-mediated renal vasoconstriction	Approved; comparative effectiveness trials	Lower risk of major adverse kidney events vs saline^23^

## Clinical applications and related investigations

The goal of developing MRFs is to create universal resuscitation fluids that are adaptable to specific medical conditions and can be modified according to a patients’ physiological status as circulating blood volume, oxygen delivery, or coagulation factors. An ideal MRF should have following features: relatively longer room temperature shelf life, more effective than current standards of crystalloids and colloids resuscitation for severe hypovolemia and related pathological conditions as coagulopathy, metabolic disturbances, and improvement in survival. MRFs can potentially be very useful in following scenarios.

### Traumatic brain injury and hemorrhagic shock

This application is the so called “holy grail” application for MRFs. Trauma-related death is usually caused by traumatic brain injury and hemorrhagic shock. The coexistence of both conditions will subject patients to higher mortality and worsened coagulopathy than patients with hemorrhagic shock alone.^4^ The management of both conditions can sometimes be paradoxical. Hemorrhagic shock is best managed with limited-volume resuscitation to minimize further blood loss and facilitate hemostasis, while traumatic brain injury may worsen with hypotension/hypovolemia in a time- and dose-dependent manner,^4^ an ideal resuscitation would support hemostasis to limit hemorrhage, increase cardiac output, and maintain vascular tone to optimize cerebral perfusion. If this could be achieved in a low-volume MRF, the portability of MRFs could potentially make it more accessible when large volume fluid and human allogeneic blood not available as in remote areas and natural disasters.^4^ St John et al. tested cocktail-style MRFs in Yorkshire swine model with traumatic brain injury, hemorrhagic shock, aortic tear and femur fracture. The fluid resuscitation was started when plasma lactate level reached 3–4 mmol/L. The experimental animals were then randomized to five groups with all groups received hydroxyethyl starch solution and vasopressin. Low- and high-dose fibrinogen groups additionally received 100 or 200 mg/kg fibrinogen respectively. A third group received tranexamic acid and low-dose fibrinogen. Two control groups only received albumin, with one group also receiving tranexamic acid. They found that blood loss was lowered and vital organ blood flow was improved with low- and high-dose fibrinogen when compared to albumin controls, but animal survival was not improved. No additional benefit of high- versus low-dose fibrinogen was observed on blood loss or survival. Tranexamic acid alone also decreased blood loss while no survival benefits. The combination of tranexamic acid with fibrinogen did not seem to offer additional benefit. Thus, the authors believe that low-volume cocktail-style MRFs containing hydroxyethyl starch, vasopressin, and fibrinogen concentrate improved outcomes when compared to control groups during limited resuscitation of polytrauma.^13^

### Septic shock

Septic shock develops from an uncontrolled inflammatory response to an infection, leading to widespread increase of capillary permeability and vasodilation, which causes a significant fluid leak into tissues and leads to a sharp blood pressure decrease. This is often accompanied by hypercoagulable state leading to microthrombi, consequently to poor tissue perfusion, hypoxemia, and organ dysfunction/failure, because the circulatory system is unable to deliver adequate blood and oxygen to meet metabolic demands.^24^ The pathophysiological alterations include Initial infection and inflammatory response: the body fights an infection (bacterial, viral, or fungal), but the immune response becomes dysregulated. Inflammatory mediators like cytokines (e.g., TNF-α, IL-1) are released, causing widespread inflammation; Vascular changes are nitric oxide-mediated vasodilation and increased capillary permeability, leading to intravascular fluid exodus and decreased peripheral resistance and hypotension; Cardiovascular effects include initial hyperdynamic phase (higher cardiac output with low systemic vascular resistance) and later hypodynamic phase (myocardium is directly damaged by inflammatory molecules and oxidative stress, leading to lower cardiac output and significant decreased in blood pressure); Coagulation cascade can exhibit as hypercoagulability (the coagulation system is activated by inflammation leading to widespread microthrombi formation in small blood vessels), and disseminated intravascular coagulation (DIC): The widespread coagulation can massively consume platelets and clotting factors, paradoxically leading to both clotting and bleeding; Organ damage will occur due to fluid leakage and poor organ blood perfusion-induced organ dysfunction; and mean cells don’t receive enough oxygen, leading to organ dysfunction. Because this process is systemic, multiple organ failure can occur simultaneously leading to multiple organ dysfunction syndrome (MODS).^24^ MRFs could theoretically be tailored to restore vascular integrity and endothelial function, improve volume status, provide vasopressing effects to offset inflammation-induced low systemic vascular resistance, optimize oxygen transportation and ameliorate the myocardial ischemia, deliver antibiotics and antidotes to neutralize circulating inflammatory cytokines and reactive oxygen species.^3^, ^4^, ^13^ Currently no literature was found available related to application of MRFs in the management of septic shock.

### Myocardial infarction and stroke

Both myocardial infarct and stroke are essentially caused by inadequate oxygen supply to the heart and brain tissues, which can be the results of vascular problems, atherosclerosis, thrombosis, embolization, vascular rupture, exterior compression or severe anemia-related hypoxemia. Systemic inflammation is increasingly recognized as a critical factor in the pathogenesis of both myocardial infarct and stroke. Inflammatory mediators contribute not only to the formation and destabilization of atherosclerotic plaques, but also induce endothelial dysfunction, thrombogenicity, and vascular reactivity.^25^ C-reactive protein is an acute-phase reactant produced by the liver in response to interleukin-6 (IL-6). C-reactive protein has been found to be elevated in both acute ischemic stroke and myocardial infarct patients. Increased C-reactive protein levels are associated with worse outcomes in both conditions and may predict future vascular events.^26^ The cytokines such as tumor necrosis factor-alpha (TNF-α) and interleukin-1 beta (IL-1β) also play an important role in propagating vascular inflammation and increasing plaque vulnerability.^27^ Endothelial dysfunction, marked by reduced nitric oxide bioavailability and increased oxidative stress, impairs the protective barrier function of the endothelium. This dysfunction sets the stage for leukocyte adhesion, vascular smooth muscle proliferation, and eventual thrombosis.^28^ MRFs with oxygen therapeutic agents could potentially ameliorate the oxygen supply issues associated with myocardial infarct and stroke. HBOCs have been tested to be added to MRFs to make the MRFs oxygen-carrying resuscitation fluids, which might have therapeutic advantages because their smaller molecules to access the inaccessible arterioles/capillaries by normal red blood cells.^4^, ^29^ Seemingly there is no literature available which investigated the application of PBOCs in MRFs.

### Major surgery

**Fig. 1:**
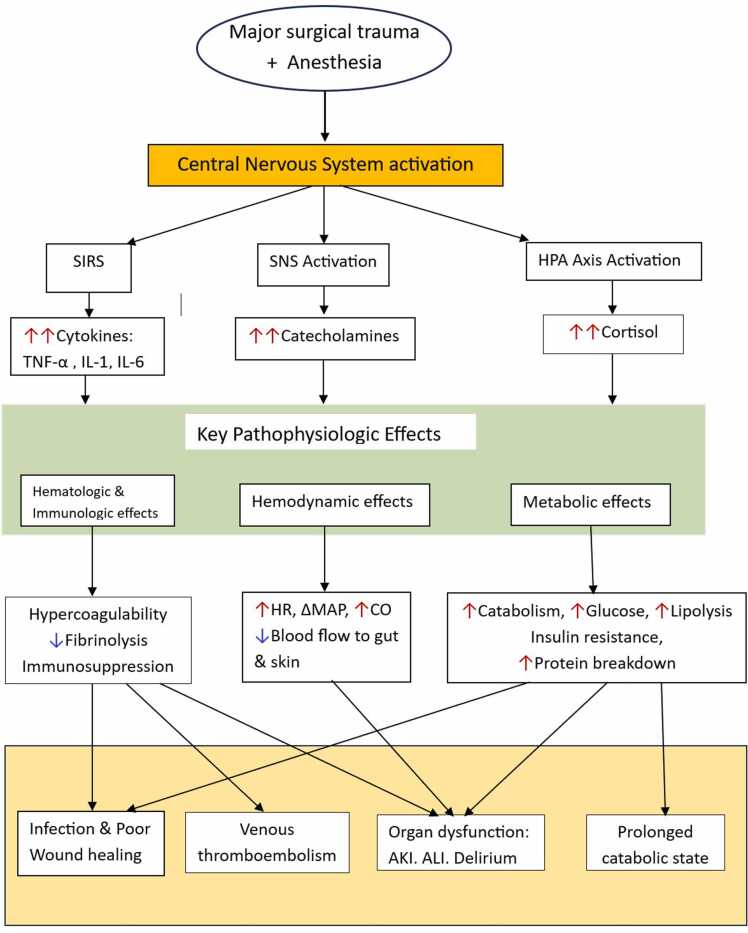
Pathophysiology of major surgery. SIRS: Systemic inflammatory response syndrome; SNS: Sympathetic nervous system; HPA: Hypothalamus-Pituitary-Adrenal Axis; TNF: Tumor necrotizing factor; IL: Interleukin; HR: Heart rate; MAP: Mean Arterial Pressure; CO: Cardiac output; AKI: Acute kidney injury; ALI: Acute lung injury.

The pathophysiology of major surgery involves multiple pathophysiological alterations (Fig. 1). Surgical stress response: The body’s reaction to surgery-related injury involves both an inflammatory and an endocrine response. Inflammatory responses can be triggered by the physical trauma which induces the release of inflammatory mediators, and the inflammatory responses can impact various organs. Endocrine response is manifested as releases of hormones like cortisol, antidiuretic hormone, and aldosterone, leading to effects like fluid retention (conserving sodium and water) and potential shifts in potassium levels. Multi-organ effects: the surgical stress can affect multiple organ systems leading to complications, such as cardiopulmonary dysfunction, gastrointestinal paralysis and dysfunction, cerebral dysfunction, increased risk of infection and blood clots (thromboembolic complications), indirect cellular injury due to factors like reduced blood supply or oxygen delivery during and after surgery.^30^ MRFs can have components targeting above pathophysiological disturbances by suppression of stress responses, anti-inflammatory, protection of vital organs via maintaining hemodynamics and enhancing oxygen delivery, and minimizing allogeneic blood transfusions in major emergency or elective surgeries.^3^, ^4^, ^5^, ^13^

## Multifunctional resuscitation fluids: advantages and disadvantages

MRFs are advanced solutions designed to go beyond simple volume expansion to address the multiple pathophysiological derangements caused by conditions such as severe hemorrhage or shock. They typically combine characteristics like oxygen-carrying capacity, clot-promoting agents, and infection-fighting properties. MRFs come with significant theoretical advantages and disadvantages. Advantages include that MRFs addresses multiple needs with one product, more convenient to use, and more readiness when in urgent/emergency situations, reduces risks of blood transfusion-related complications. MRFs are novel alternatives to traditional allogeneic blood transfusions which carry significant risk of complications. MRFs can potentially replicate functions of whole blood, offering oxygen delivery and clotting support without the drawbacks of relying on donated blood (Table 2).^31^

**Table 2:** Multifunctional resuscitation fluids: advantages and disadvantages.

**Advantage**	**Disadvantage**
Targeted therapy: Addresses multiple pathological conditions simultaneously, not just the hemodynamic deficit.	Immense complexity: formulation is exponentially more complex than saline, raising manufacturing and quality control challenges.
Reduced secondary injury: potential to attenuate ischemia-reperfusion injury, MODS, and nosocomial infections.	Cost prohibitive: current production costs are orders of magnitude higher than traditional fluids, limiting widespread adoption.
Logistical Benefit: Universal compatibility, longer shelf-life, and no refrigeration required versus packed red blood cells.	Safety and immunogenicity: novel components (PEG, synthetic lipids) carry a risk of unexpected immune reactions (e.g., complement activation-related pseudo-allergy).
Synergistic Effects: A single infusion achieves what would require multiple separate drug administrations.	Narrow therapeutic window: over-dosing a pharmacologically active fluid could have unintended consequences (e.g., excessive immunosuppression).

MODS: multiple organ dysfunction syndrome, PEG: Polyethylene Glycol.

MRFs, developed to address multiple pathological conditions/issues like hemorrhage, hypotension, inflammation, and cellular dysfunction simultaneously, provides multiple theoretical and practical advantages but also carry significant risks and disadvantages. MRFs may cause complex and sometimes contradictory effects that are difficult to manage.^32^ MRFs related fluid overload and subsequent organ tissue edema leads to fluid leaking into the tissues, causing edema in the lungs (pulmonary edema), intestines (gut edema), and other areas. Pulmonary edema can impair gas exchange in the lungs, interfere with organ function, and increase the risk of infection. Dilutional coagulopathy and electrolyte imbalances can also occur. Some balanced solutions contain potassium, which can cause hyperkalemia (high potassium) in patients on specific medications or with kidney disease. Other fluids can contribute to hyponatremia (low sodium).^32^ MRFs may also cause cellular and systemic toxicity such as reperfusion injury, inflammatory response, and nephrotoxicity. Synthetic colloid solutions, like hydroxyethyl starch, have been shown to increase the risk of acute kidney injury and may worsen kidney function.^27^ Unpredictable response, regulatory and safety concerns, and cost and accessibility are also potentially their disadvantages.

## Current status and challenges

The development of cocktail-style MRFs is still in its early stages, with most research focused on animal experiments, preclinical and small-scale clinical studies. Despite some promising data from the preliminary investigations, significant hurdles remain. Clinical safety and efficacy in rigorous clinical trials are needed to demonstrate that new products are both safe and effective. Past safety signals, such as vasoconstriction and renal injury from earlier HBOCs, highlight the need for caution. MRFs may be used for emergency and pre-hospital scenarios, they must be affordable, easy to transport, and storable for long periods. These factors could limit the initial adoption of some advanced complex products. Currently no validated, rapid biomarkers to identify which patients will benefit from a specific MRF versus those who will do well with standard care. Scaling up the synthesis of uniform, stable nanoparticles and liposomes for global use remains a formidable engineering challenge. The regulatory pathway for complex multifunctional fluids is challenging and requires a clear understanding of their biological mechanisms and potential side effects.^27^, ^33^, ^34^ Unsurprisingly, as of now, there are no widely accepted commercial formulations of these fluids in standard medical practice. The next steps in research will likely focus on large-scale clinical trials to evaluate the safety and efficacy of these MRFs across diverse patient populations with specific cocktail combinations for specific clinical scenarios. There is also a need for more robust studies on the long-term effects of MRFs, as well as their impact on survival, recovery, and quality of life in critically ill patients. Additionally, advances in personalized medicine could help tailor fluid therapy based on genetic, physiological, and inflammatory biomarkers, potentially improving patient outcomes.^34^

## Future directions

The future directions for MRFs are moving toward more advanced, targeted, and personalized therapies to improve oxygen delivery, reverse coagulopathy, and mitigate inflammation. While traditional crystalloid and colloid fluids primarily expand volume, next-generation multifunctional fluids are designed to address multiple consequences of severe blood loss, shock, inflammation and coagulopathy simultaneously.^35^

The path forward for MRFs involves developing point-of-care diagnostics to match the right MRFs to the patient’s specific hemodynamic, metabolic status and shock phenotype (e.g., hyperinflammatory vs. endothelial leak dominant),^34^, ^35^ creating increasingly sophisticated platforms that mimic natural cellular components, such as advanced oxygen carriers, platelet-like particles for hemostasis or neutrophil-derived vesicles for anti-microbial activity, designing multifunctional fluids that release their cargo (e.g., hemostatic agents, oxygen, drug) only in response to specific pathological triggers like low pH or elevated protease activity in the shock microenvironment,^34^ and intensifying research into low-cost, thermostable MRFs for use in resource-limited settings and disaster scenarios.^36^

## Conclusions

The concept of cocktail-style multifunctional resuscitation fluids represent an exciting advancement in fluid therapy, offering the potential to improve survival and reduce organ damage/dysfunction in critically ill patients. While preclinical and clinical studies have shown promise, there are still significant hurdles and challenges to overcome. Further research will be needed to optimize formulations, address safety concerns, and ensure that these fluids can be effectively incorporated into clinical practice. While the challenges of cost, regulation, and manufacturing are substantial, the promise of transforming a supportive therapy into a definitive, injury-modifying treatment is too great to ignore. The coming decades will be defined by rigorous experimental and clinical validation studies, along with the collaborative effort needed to overcome these barriers, ultimately paving the way for a new standard of care in critical illness.

## CRediT authorship contribution statement

**Frank Chen:** Validation, Writing – review & editing. **Henry Liu:** Writing – review & editing, Conceptualization. **Lauren C. Hollifield:** Writing – review & editing, Data curation. **Geoffrey L. Liu:** Writing – review & editing, Writing – original draft, Conceptualization. All authors have read and agreed to the published version of the manuscript.

## Consent for publication

Not applicable.

## Ethical statement

Not applicable.

## Funding

This research received no external funding.

## Declaration of competing interest

The authors declare that they have no known competing financial interests or personal relationships that could have appeared to influence the work reported in this paper. Henry Liu is the Editor-in-Chief of *Journal of Anesthesia and Translational Medicine* and was not involved in the editorial review or the decision to publish this article.

## Data Availability

All data cited in this review are publicly available through the original publications listed in the references. Source data for figures are provided with this paper.
